# Not just disclosure of generative artificial intelligence like ChatGPT in scientific writing: peer-review process also needs

**DOI:** 10.1097/JS9.0000000000001619

**Published:** 2024-05-09

**Authors:** Haiyang Wu, Wanqing Li, Xiaofeng Chen, Cheng Li

**Affiliations:** a Department of Orthopaedics, The First Affiliated Hospital of Zhengzhou University, Zhengzhou; b Department of Clinical College of Neurology, Neurosurgery and Neurorehabilitation, Tianjin Medical University, Tianjin; c Department of Operating Room, Xiangyang Central Hospital, Affiliated Hospital of Hubei University of Arts and Science, Xiangyang; d Department of Orthopaedic Surgery, Yangxin People’s Hospital, Yangxin, Hubei; e Department of Orthopaedic Surgery, Beijing Jishuitan Hospital, Capital Medical University, Beijing, People’s Republic of China; f Center for Musculoskeletal Surgery (CMSC), Charité-Universitätsmedizin Berlin, Corporate Member of Freie Universität Berlin, Humboldt University of Berlin, Berlin Institute of Health, Berlin, Germany

*Dear Editor,*

The Chatbot Generative Pretrained Transformer (ChatGPT), an artificial intelligence (AI)-powered prominent large language models (LLMs) developed by OpenAI, demonstrates an exceptional proficiency in comprehending and generating natural language text. Its capacity to engage in human-like dialogues has garnered widespread attention from both the public and academia, and multiple journals including *International Journal of Surgery* have published several papers on ChatGPT recently^1,2^. Many scholars have begun to focus on the potential applications of ChatGPT in the biomedical field, especially in scientific research writing. The trend of employing ChatGPT in the realm of scholarly writing has become increasingly conspicuous, as evidenced by past research. Its multifaceted functionalities encompass topic selection, outline composition, discourse elucidation, stylistic transformation, expression refinement, and content summarization, all of which contribute synergistically to enhancing the quality and efficiency of writing endeavors. Moreover, in order to distinguish ChatGPT-generated content from human-authored text, an increasing number of journals are instituting disclosure guidelines pertaining to the utilization of generative AI, like ChatGPT. They suggest that authors utilizing generative AI should maintain transparency and furnish information regarding the modus operandi of employing such technology in sections such as research methodology, acknowledgments, cover letters, or other segments. Nevertheless, despite the majority of journals instituting guidelines regarding the use of ChatGPT in scientific writing, they often overlook another crucial stage in scientific publication: the peer-review process.

A recent study has found that within the peer-review texts of several top AI conferences including ICLR 2024, NeurIPS 2023, CoRL 2023, and EMNLP 2023, 6.5–16.9% may have undergone substantial modifications by LLMs, surpassing the scope of mere spell-checking or minor writing updates^3^. This finding suggests that ChatGPT is not merely utilized in scientific writing, but peer-review experts may also be employing ChatGPT. Hosseini *et al*.^4^ has investigated the potential impact of using ChatGPT on the five core themes within discussions about peer-review including reviewers’ role, editors’ role, functions and quality of peer reviews, reproducibility, and the social and epistemic functions of peer reviews. Their findings showed that ChatGPT possesses the capacity to significantly transform the functions performed by both peer reviewers and editors within academic publishing. By aiding both parties in the expeditious composition of insightful evaluations or decisive correspondence, ChatGPT serves as enablers for enhancing the caliber of peer-review processes while also mitigating concerns stemming from reviewer scarcity. Another study has compared the assessment results of 21 research articles among two humans, ChatGPT 3.5 and ChatGPT 4. Their results show that the subjective review opinions given by human reviewers and ChatGPT, especially ChatGPT 4, are similar^5^.

Therefore, these studies suggest that the use of ChatGPT in the peer-review process seems feasible. Reviewers could generate a clear review report by inputting their own notes into ChatGPT, and the opportunity to streamline the review process may be enough to encourage reviewers to accept the invitation. Yet the opacity of generative AI’s training data, inner workings, data processing, and development processes raises concerns about potential bias, confidentiality, and reproducibility of review reports. In May 2023, the International Committee of Medical Journal Editors (ICMJE) released the latest recommendations on the use of AI in the peer-review process. The recommendation states that reviewers should not upload manuscripts to software or other AI technology platforms that cannot guarantee confidentiality. Second, reviewers should disclose to the journal whether and how AI technology is used in the process of evaluating manuscripts or writing reviewer comments. Subsequently, numerous publishers such as *BMJ* and *Science* also implemented comparable peer-review policies.

However, after browsing the official websites of the top five journals (*JAMA Surgery*, *International Journal of Surgery*, *Journal of Neurology Neurosurgery and Psychiatry*, *Annals of Surgery*, *British Journal of Surgery*) with the highest impact factors in the field of surgery, although all journals mentioned disclosing the use of generative AI during the paper submission process, only two mentioned the use of generative AI during the peer-review process. JAMA Surgery stated that reviewers are instructed not to submit confidential manuscripts, abstracts, or other text into a chatbot, language model, or similar tool. The *Journal of Neurology Neurosurgery and Psychiatry* suggests that if reviewers use AI technology to improve word processing and language, they should declare this when submitting their reports. However, reviewers should preserve the confidentiality of the peer-review process by not putting unpublished manuscripts into publicly available AI tools. The results suggest that disclosure of generative AI use during the peer-review process does not attract enough attention. The utilization of generative AI entails a plethora of ethical and legal quandaries, encompassing issues such as data privacy and copyright protection. These matters warrant comprehensive scrutiny and discourse within the peer-review process. Advocating for heightened transparency regarding the implementation of generative AI serves to propel advancements and enhancements within the peer-review process, fostering a deeper comprehension and utilization of this technology within academia.

## Ethical approval

This study does not include any individual-level data and thus does not require any ethical approval.

## Sources of funding

This study is supported by China Postdoctoral Science Foundation (2022M720385) and Beijing JST Research Funding (YGQ-202313).

## Author contribution

H.W.: conceptualization, methodology, data curation, formal analysis, resources, investigation, and writing – original draft; W.L.: conceptualization, methodology, data curation, formal analysis, resources, and investigation; X.C.: conceptualization, methodology, data curation, formal analysis, resources, and investigation; C.L.: methodology, data curation, formal analysis, resources, investigation, and writing – review and editing.

## Conflicts of interest disclosure

The authors declare no conflicts of interest.

## Research registration unique identifying number (UIN)

1. Name of the registry: not available.
2. Unique identifying number or registration ID: not available.
3. Hyperlink to your specific registration (must be publicly accessible and will be checked): not available.

## Guarantor

Haiyang Wu and Cheng Li.

## Data availability statement

The data underlying this article will be shared by the corresponding author on reasonable request.
